# Immunoregulation in Fungal Diseases

**DOI:** 10.3390/microorganisms4040047

**Published:** 2016-12-10

**Authors:** Jonathan A. Roussey, Michal A. Olszewski, John J. Osterholzer

**Affiliations:** 1 Division of Pulmonary and Critical Care Medicine, Department of Internal Medicine, University of Michigan Health System, Ann Arbor, MI 48109, USA; olszewsm@med.umich.edu (M.A.O.); oster@med.umich.edu (J.J.O.); 2 Pulmonary Section, Medical Service, VA Ann Arbor Health System, Ann Arbor, MI 48105, USA; 3 Graduate Program in Immunology, University of Michigan Health System, Ann Arbor, MI 48109, USA

**Keywords:** immunoregulation, fungi, dendritic cell (DC), regulatory T cell (Treg cell), programmed cell death 1 (PD-1), cytotoxic T lymphocyte-associated protein 4 (CTLA-4), interleukin-10 (IL-10), protective tolerance, immune reconstitution inflammatory syndrome (IRIS)

## Abstract

This review addresses specific regulatory mechanisms involved in the host immune response to fungal organisms. We focus on key cells and regulatory pathways involved in these responses, including a brief overview of their broader function preceding a discussion of their specific relevance to fungal disease. Important cell types discussed include dendritic cells and regulatory T cells, with a focus on specific studies relating to their effects on immune responses to fungi. We highlight the interleukin-10, programmed cell death 1, and cytotoxic T lymphocyte-associated protein 4 signaling pathways and emphasize interrelationships between these pathways and the regulatory functions of dendritic cells and regulatory T cells. Throughout our discussion, we identify selected studies best illustrating the role of these cells and pathways in response to specific fungal pathogens to provide a contextual understanding of the tightly-controlled network of regulatory mechanisms critical to determining the outcome of exposure to fungal pathogens. Lastly, we discuss two unique phenomena relating to immunoregulation, protective tolerance and immune reactivation inflammatory syndrome. These two clinically-relevant conditions provide perspective as to the range of immunoregulatory mechanisms active in response to fungi.

## 1. Introduction

Fungal infections present unique challenges for the host immune system. Some fungi, such as *Candida albicans* (*C. albicans*), exist as commensals and in many cases are beneficial to the host by limiting overgrowth of other potentially harmful microorganisms [1]. Many fungi are environmental saprophytes generally harmless to immunocompetent mammalian hosts, although immunosuppression, long-term antibiotic treatment, and corticosteroid treatment can result in opportunistic infections with several species including *C. albicans*, *Cryptococcus neoformans* (*C. neoformans*), *Histoplasma capsulatum* (*H. capsulatum*), and *Aspergillus fumigatus* (*A. fumigatus*), amongst others [2]. Nosocomial and environmentally acquired fungal infections within seemingly healthy individuals are increasingly common [3]; currently unrecognized immune defects may account for infections in these individuals as has been recently shown for patients with cryptococcal meningitis discovered to have auto-antibodies against Granulocyte-Macrophage Colony Stimulating Factor [4,5].

**Figure 1:**
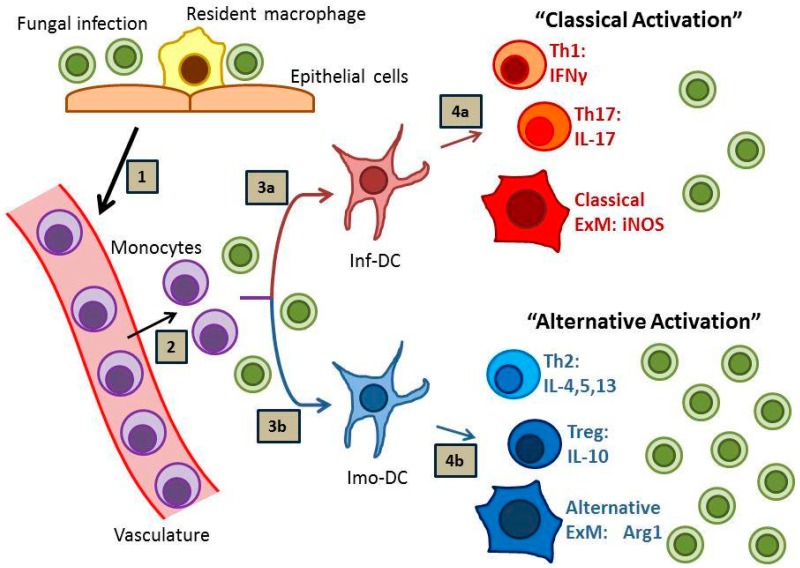
Immunity to Fungal Infections. Fungal infection (1) stimulates the arrival of monocytes at the site of infection (2), which subsequently mature into monocyte-derived dendritic cells (moDCs). Depending on local environmental host and pathogen factors, these moDCs can develop into either inflammatory DCs (inf-DCs; 3a) or immunomodulatory DCs (imo-DCs; 3b), which subsequently direct the immune response. Inf-DCs promote sterilizing immunity characterized by interferon gamma (IFNγ)-producing Th1 cells, interleukin (IL)-17-producing Th17 cells, and “classically activated” exudate macrophages (ExMs; 4a). Imo-DCs promote fungal persistence characterized by IL-10-producing Treg cells, IL-4-, IL-5, and IL-13-producing Th2 cells, and “alternatively-activated” exudate macrophages (4b).

Effective host defense against fungal disease is heavily reliant on the adaptive arm of the immune system, which is strongly influenced by local interactions between antigen presenting cells, especially dendritic cells (DCs), and subsets of cluster of differentiation (CD) 4 (CD4)^+^ T cells. Sterilizing immunity against fungal pathogens most strongly correlates with the development of “inflammatory” DCs (or “DC1”), Th1 and Th17 immune responses and the subsequent classical activation of effector macrophages (or “M1”) (Figure 1, pathway 1) [6,7,8]. In contrast, many fungal infections prove difficult to eradicate and provoke “immunomodulatory” DC (or “DC2”), Th2 and T regulatory (Treg) responses, and local alternative macrophage activation (or “M2”) with resultant persistent infection (Figure 1, pathway 2) [9,10,11,12]. In the absence of either response, as in the case of HIV-AIDS or potent immunosuppression, progressive disease may develop which is often lethal to the host.

**Figure 2:**
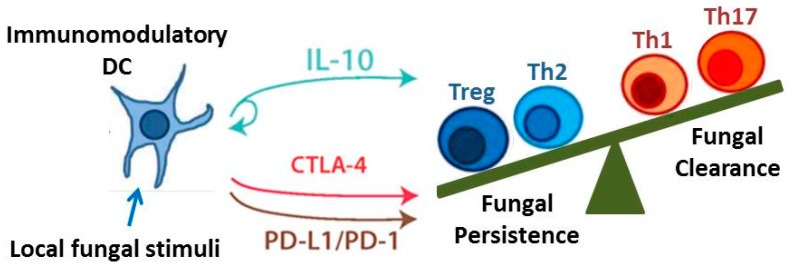
Immunoregulatory Pathways. The development of immunomodulatory DCs leads to regulatory signaling via the IL-10, programmed cell death protein (PD)-1, and cytotoxic T lymphocyte-associated protein (CTLA)-4 pathways. These pathways in turn promote the development of Treg cells (as well as Th2 cells) and inhibit the development of Th1 and Th17 cells. The balance of these immunoregulatory pathways may determine whether persistent infection results in chronic immune-mediated tissue destruction (as occurs in Allergic Bronchopulmonary Mycosis) or Protective Tolerance.

The objective of this review is to identify and discuss key immune cells and immunoregulatory signaling pathways that critically contribute to the outcome of fungal infections. Although there are numerous immunoregulatory mechanisms currently under investigation, we highlight those that are particularly well-researched and may yield promising novel therapeutics. We specifically focus on DCs and regulatory T cells given their capacity to orchestrate and influence the function of numerous effector T cell and macrophage subsets. We also identify three pathways: Interleukin-10 (IL-10) signaling, the programmed cell death protein-1 (PD-1) signaling pathway, and the cytotoxic T lymphocyte-associated protein 4 (CTLA-4) signaling pathway, as examples of important immunoregulatory mechanisms and checkpoints that modulate adaptive immune responses (Figure 2). Lastly, we discuss two unique and opposing phenomena that demonstrate the range of possible regulatory conditions (or lack thereof) that can arise when the immune system interacts with fungi. Protective tolerance represents a containment strategy implemented by the host to “cohabitate” with a pathogenic fungus rather than risk extensive damage due to attempts at sterilizing immunity. In contrast, immune reconstitution inflammatory syndrome (IRIS) is a condition that may occur when anti-fungal immunity is rapidly restored, resulting in an over-exuberant inflammatory response detrimental to the host. Collectively, our coverage of these topics highlights the diversity and interrelatedness of immunoregulatory mechanisms that influence the outcome of fungal infection.

## 2. Immunoregulatory Cells

### 2.1. Dendritic Cells

As a link between innate and adaptive immunity, dendritic cells (DCs) have an inherent role in regulating immunity through their ability to influence initial T cell responses [6] to a wide array of antigenic stimuli including those originating from fungi and other pathogens, tissue grafts, and cancerous cells. Factors broadly influencing the outcome of DC: T cell interactions include the origin of the antigen and the antigenic load; evidence suggests a low antigenic load contributes to Th2 immunity whereas a high antigenic load promotes Th1 responses [13]. The outcome of these interactions is further influenced by the specific subset and microenvironmental location of the DCs participating in the response. Dendritic cells most relevant to fungal infection include plasmacytoid DCs (pDCs) that reside in secondary lymphoid organs (including lymph nodes) and circulate in the blood, conventional DCs (cDCs), subsets of dermal DCs (dDCs), and monocyte-derived DCs (moDCs) that migrate to sites of infection from bone marrow via the blood.

Our understanding of the potential regulatory capabilities of plasmacytoid DCs (pDCs) is evolving. Plasmacytoid DCs have a well-defined role in mediating antiviral immunity [14,15], can induce Treg cells [16,17] and regulate both Treg cells and Th17 cells at mucosal surfaces [18]. In the process, pDCs can modulate the balance between CD4^+^ and CD8^+^ T cell responses to certain viral and bacterial infections [18] and can mediate tolerance to tissue grafts [19]. Evidence suggests pDCs may similarly regulate innate and adaptive immune responses to fungal pathogens. Specifically, Ramirez-Ortiz and colleagues demonstrated that pDCs recognize and inhibit the growth of *A. fumigatus* in vitro [20]; their additional in vivo studies demonstrate that pDCs accumulate in the lungs of *A. fumigatus*-infected mice and that pDC depletion enhances fungal growth [21]. This finding, combined with evidence suggesting a role in regulating T cell differentiation and activity [22], suggests that pDCs modulate immunity to fungi, especially at mucosal surfaces.

Conventional DCs (cDCs) include populations of tissue-resident DCs likely to first encounter fungal pathogens at mucosal surfaces including the skin. Two dominant subsets can be identified in most peripheral tissues: CD103^+^CD11b^−^ cDCs (CD103^+^ cDCs) expressing the transcription factor Batf3 and CD103^−^CD11b^+^ cDCs (CD11b^+^ cDCs) expressing the transcription factor interferon regulatory factor (IRF)4. Although best known for their immunostimulatory properties, both subsets have immunoregulatory activity under certain circumstances. CD103^+^ cDCs can induce peripheral tolerance to self-antigens by inducing apoptosis in self-reactive T cells [23] and generally participate in regulatory activity by secreting the immunomodulatory cytokine transforming growth factor beta (TGFβ) during steady-state conditions [24], helping to maintain tolerance during times of health. CD11b^+^ cDCs can regulate autoimmune responses including those active in rheumatoid arthritis [25] and autoimmune diabetes [26]. Dermal dendritic cells (dDCs) residing in the skin include CD103^+^ cDCs, CD11b^+^ cDCs, and also Langerin^+^ dDCs [27]. The location of dDCs in the skin makes them prone to frequent exposure to fungi. In a murine model of cutaneous *C. albicans* exposure, CD103^+^ dDCs generate IL-12 and promote Th1 cell differentiation whereas CD11b^+^ DCs generate high amounts of IL-1β and to a lesser extent IL-6 and IL-12 [28]. In contrast, Langerin^+^ dDCs demonstrated the most robust immunoregulatory role by blunting Th17 responses [28]. In this model, DC responses are further modulated by fungal morphology [29], thus demonstrating highly selective immunoregulatory properties for different subsets of dDCs in response to differing forms of the same fungal pathogen.

Monocyte-derived DCs (moDC) are important modulators of ongoing immune responses within infected microenvironments. In the lung, these cells are derived from newly recruited C-C chemokine receptor type 2 (CCR2)^+^ Lymphocyte antigen C (Ly6C)^high^ bone marrow monocytes. In our studies using murine models of protective immunity against cryptococcal lung infection in Bagg Albino (BALB/c) mice, we have demonstrated that moDCs are proinflammatory and promote potent Th1 and Th17 responses [30,31,32] (refer to Figure 1, pathway 1). Mice lacking CCR2 display impaired accumulation of moDCs, develop signs of Th2-type immunity [31], and fail to clear the fungus from the lungs [33]; similar findings have been observed for CCR2-deficient mice infected with *H. capsulatum* [34]. Depletion of CCR2^+^ Ly6C^high^ monocytes in a murine model of *A. fumigatus* infection resulted in reduced fungal transport to draining lymph nodes, diminished CD4^+^ T cell priming, and inhibited fungal clearance [35], further implicating monocyte-derived dendritic cells as critical regulators of immunity. However, despite the proinflammatory potential of moDCs, in vitro studies have shown that human and murine monocytes and DCs express high amounts of the immunomodulatory cytokine IL-10 in response to *C. neoformans* antigens [36,37,38] (and unpublished results). This finding likely has significant in vivo relevance as evidenced by studies using a murine model of persistent pulmonary *C. neoformans* infection in C57BL/6 mice; in these studies which utilized infected IL-10^−/−^ mice or C57BL/6 mice treated with antibody-mediated blockade of IL-10 signaling, IL-10 and the development of immunomodulatory moDCs (or “imo-DCs”; refer to Figure 1, pathway 2) were clearly implicated in the suppression of Th1 and Th17 responses, reductions in macrophage activation, and impairments in fungal clearance [9,39]. In addition to IL-10 production, moDCs also upregulate Programmed cell death protein ligands 1 and 2 (PD-L1 and PD-L2) in response to cryptococcal mannoprotein in vitro and in response to persistent cryptococcal infections in vivo (unpublished observations); further discussion of the IL-10 and PD-1 signaling pathways can be found later in this review. Collectively, these data contribute to an emerging paradigm identifying moDCs as highly adaptable cells (i.e., “plastic”), which can promote either proinflammatory or immunoregulatory responses within infected or injured peripheral tissues depending on the collective context of local microenvironmental cues. Determining what factors promote inflammatory or immunomodulatory DC phenotypes represents an area of intense investigation beyond the scope of this review.

As outlined in this section, by bridging innate and adaptive immune responses, DCs are thus critically positioned to regulate immunity against fungal pathogens. In the following sections, we will further highlight specific immunoregulatory mechanisms influenced by DCs including the development of regulatory T cells and the modulatory effects mediated by the IL-10, PD-1, and CTLA-4 signaling pathways.

### 2.2. Regulatory T Cells

Regulatory T cells (Treg cells), broadly defined as CD4^+^CD25^+^ T cells expressing the transcription factor forkhead box protein 3 (FoxP3), were named to reflect their capacity to down-regulate immune responses to a wide variety of self and foreign antigens [40,41,42,43,44,45], as well as promote homeostasis [46] and transplantation tolerance [47]. While acknowledging the complexity of this field in terms of the numerous subsets and functions of Treg cells, in this review, our generalized use of the term Treg cell includes subsets of both natural Treg cells (nTreg) that develop in the thymus and primarily serve to prevent autoimmune reactions [48,49,50], and induced Treg cells (iTreg), which develop in the periphery in response to persistent antigen exposure [51]. Treg cells exert their regulatory effects largely through production of IL-10 and TGFβ, although contact-dependent mechanisms are also present [48,52,53,54]. There are many proposed subsets of Treg cells with unique functions and phenotypes; such information is beyond the scope of this discussion and we refer the reader to several excellent reviews on the subject [48,49,50,55,56]. As alluded to in the preceding section, we also highlight evidence that DCs prominently influence the development of numerous subsets of Treg cells, including both the natural and induced varieties [57].

There is abundant evidence suggesting a functional role for Treg cells in the context of fungal disease with numerous reports suggesting that Treg cells enhance protective immunity [58,59,60,61,62,63,64] whereas in other models, Treg cells promote fungal dissemination and immunopathology [65,66,67]. Studies performed in mice infected with *C. albicans* have proven particularly informative as numbers of Treg cells markedly increase in infected mice (relative to uninfected control mice) [65,68,69]. These Treg cells were shown to modulate immune responses of the organism by inhibiting Th1 and Th2 activity while promoting Th17 responses [58,65,67,68,69]. The enhancement of Th17 cell abundance was in part attributable to Treg cell consumption of IL-2 [58,65] consistent with the previously defined capacity of Treg cells to scavenge IL-2 via their high affinity IL-2 receptor [70]. In contrast, other studies have suggested an inhibitory effect of Treg cells on Th17 activity using the same infectious agent [71], suggesting substantial phenotypic and functional plasticity; Treg cell phenotypes are likely influenced by local tissue microenvironments as Th17 responses were inhibited in a gastro-intestinal model of *C. albicans* infection whereas they were enhanced in a disseminated infection model [58,71].

Molecules modulating Treg cell development and trafficking have been shown to impact the involvement of Treg cells in fungal immunity. Specifically, Toll Like Receptor 2 (TLR2) promotes Treg cell development during *C. albicans* infection, as evidenced by the reduction in Treg cells and lower levels of the immunoregulatory cytokine IL-10 observed in infected TLR2^−/−^ mice that display increased resistance to disseminated candidiasis [59]. In support of TLR2 as a critical mediator of Treg activity, the absence of TLR2 in a mouse model of chronic pulmonary *Paracoccidioides brasiliensis* (*P. brasiliensis*) infection leads to impaired Treg cell expansion and skewing of adaptive immunity toward a Th17 phenotype [66]. CCR5 plays a role in Treg cell migration to sites of *P. brasiliensis* infection in the lung, as loss of CCR5 results in impaired Treg accumulation and subsequently enhanced effector responses concomitant with improved granuloma formation and enhanced control of disseminated disease; adoptive transfer of Treg cells from infected wild type (WT) mice into the lungs of infected CCR5^−/−^ mice leads to enhanced disease [60]. A similar effect is seen in the context of *H. capsulatum* infection, as a reduction in Treg cell abundance in CCR5^−/−^ mice leads to improved fungal clearance following initiation of adaptive immunity [63]. Thus, Treg cell function is influenced by both local fungal sensing receptors (including TLRs) and the ability of Treg cells to migrate to sites of fungal infection.

Recent reports suggest a beneficial role for Treg cells in response to persistent pulmonary *C. neoformans* infection in C57BL/6 mice, which is characterized by strong Th2 responses that contribute to chronic allergy-mediated lung damage comparable to that observed in patients with allergic bronchopulmonary mycosis [8]. In a study by Schultze et al., the authors depleted Treg cells in mice with cryptococcal infection using DEREG (DEpletion of REGulatory T cells) mice and showed that depletion of Treg cells exacerbated Th2 responses as evidenced by increased mucus production, enhanced eosinophilia, and increased IgE production [61]. Critically, fungal burden in the lungs of DEREG mice was elevated as compared to that seen in infected WT C57BL/6 mice, demonstrating that suppression of Th2 responses enhanced protective immunity. Using *C. neoformans* peptide-specific major histocompatibility complex (MHC) II tetramers, Wiesner and colleagues further demonstrated that Treg cells accumulating in the lungs of infected mice are overwhelmingly specific for *C. neoformans* antigens, and this study provided additional evidence that Treg cells suppress detrimental Th2 immunity in response to infection [62]. The importance of maintaining balanced Treg cell capabilities was also demonstrated using an experimental model of *A. fumigatus* infection in which Treg cells were essential for both the control of the infection while simultaneously limiting excessive damage to the host [64]; we will revisit this concept of balanced immune regulation in the section on Protective Tolerance (Section 4.1). In contrast, dysregulated Treg cell activity has proven detrimental in the case of human paracoccidioidomycosis (PCM) [67], with elevated Treg cell levels seen in patients with active disease as compared to that seen in patients actively receiving treatment or healthy controls.

Collectively, these studies highlight an essential role for Treg cells in the immune regulation required to combat these complex fungal pathogens. As will be discussed later in this review, Treg cells contribute to multiple immunoregulatory processes seeking to balance the potency of effector immune responses with the potential of these same effector responses to cause collateral tissue damage. In the sections that follow, we will revisit the role of Treg cells in the carefully regulated response to fungal pathogens as we further discuss their role in the IL-10, PD-1, and CTLA-4 signaling pathways.

## 3. Immunoregulatory Signaling Pathways

### 3.1. IL-10 Signaling

Interleukin-10 is a critical mediator of immune tolerance [72] and is intimately involved in the immunoregulatory functions of DCs [39,73] and Treg cells [44,54]; both of which have the capacity for potent IL-10 production. When considering the breadth of control that these two cell types have over immunity, this identifies IL-10 as a key signaling pathway in the field of immunoregulation. IL-10 was initially described as a cytokine synthesis inhibitory factor due to its capacity to inhibit production of Type 1 cytokines [74]; subsequent studies confirm that IL-10 impairs excessive production of IL-1, IL-6, IL-23, interferon gamma (IFNγ), and tumor necrosis factor alpha (TNFα) [75,76]. Early studies demonstrated that IL-10^−/−^ mice spontaneously develop colitis [77], and a more recent study showed that the development of colitis requires microbial stimulation [75].

The role of IL-10 in promoting peripheral tolerance and in modulating immune responses to numerous infectious and non-infectious insults has since been well-characterized, and we refer the reader to several excellent reviews on the subject [78,79,80]. Our understanding IL-10 in the context of fungal infections is less developed although investigations involving the endemic fungal pathogen *C. neoformans* have proven particularly informative. Studies performed using human peripheral blood-derived monocytes and DCs exposed to cryptococcal antigens have shown these cells produce IL-10 and (or) respond to IL-10 by reducing expression of MHC II [36,37,38]. In support of these findings, others have shown that enhanced IL-10 expression correlates with disseminated cryptococcosis in patients with AIDS [81]. Murine models utilizing experimental cryptococcal lung infection in C57BL/6 mice have shown that IL-10-deficient mice display improved clearance of the organism from the lung, which coincided with a skewing of the CD4^+^ T cell polarization profiles from Th2 to Th1 predominant [9]. Specifically, IL-10 deficiency was characterized by reductions in tissue eosinophilia and expression of IL-4, IL-5, and IL-13, whereas Th1 responses (TNFα and IL-12 expression) were enhanced. Our group has since directly demonstrated that persistent cryptococcal lung infection in wild type C57BL/6 mice is associated with sustained IL-10 production by lung leukocytes [39], and we further showed that IL-10 signaling blockade (using a blocking antibody to the IL-10 receptor) reduced fungal burden and systemic dissemination even if administered after persistent infection had been established [39]. Our findings suggested that the protective effect of IL-10 signaling blockade was likely mediated through enhanced Th1 and Th17 responses and increased activation of effector macrophages [39]. Additional murine studies showing that a highly virulent strain of *C. neoformans* induces greater IL-10 expression than a less virulent strain [82] suggest that the microbe itself may alter IL-10 driven immunoregulation.

Studies investigating the role of IL-10 signaling in fungal pathogenesis are not limited to studies of *C. neoformans*. Limited data from human studies have identified polymorphisms in the IL-10 gene associated with increased susceptibility to invasive candidiasis [83], whereas polymorphisms in the IL-10 promoter have been associated with either resistance or susceptibility to invasive pulmonary aspergillosis [84]. In murine studies, IL-10 expression also occurs in response to murine infection with *C. albicans* [85], *H. capsulatum* [86] and *A. fumigatus* [87] and the absence of IL-10 is associated with improved fungal clearance. In particular, intravenous infection with *C. albicans* is quickly cleared in IL-10^−/−^ mice compared to wild-type mice, which is attributed to more efficient fungal killing by neutrophils [85]. Interestingly, IL-10 production in response to *C. albicans* infections has been linked to signaling through Toll Like Receptor 2 (TLR2) with additional downstream effects on the expansion of Treg cells [59]; depletion of Treg cells improved resistance, thereby underscoring links between the IL-10 signaling pathway and Treg cell-mediated immune modulation. 

Thus, studies to date identify the IL-10 signaling pathway as a critical contributor to the immunoregulatory networks that develop in response to fungal infections. An overabundance of IL-10 impairs fungal clearance and appears essential for the development of persistent fungal infections. Yet deficiencies in local IL-10 production may result in over-exuberant inflammation including immune reactions to commensal organisms. In the next sections, we further highlight interrelationships between immunoregulatory mechanisms as we discuss the role of the PD-1 and CTLA-4 signaling pathways in immune checkpoints.

### 3.2. Programmed Cell Death Pathway

The programmed cell death signaling pathway has rapidly gained attention as a critical immune checkpoint, initially identified for its ability to severely inhibit T cell proliferation and effector activity [88,89]. The pathway consists of the receptor PD-1 and its ligands PD-L1 and PD-L2 [90]. PD-1 is expressed on activated T cells [91,92], with evidence suggesting its expression on other cell types including B cells and macrophages [92,93]. PD-L1 is expressed on many cell types including antigen presenting cells (APCs), T cells, epithelial cells, and is often upregulated during inflammation. PD-L2 expression is primarily restricted to APCs such as DCs and macrophages [94].

Similar to IL-10, PD-1 plays a prominent role in maintaining peripheral tolerance, and several studies note that elimination of this pathway leads to the development of autoimmune disorders in animal models, with PD-1 signaling-deficient mice developing conditions including Lupus-like proliferative arthritis and autoimmune dilated cardiomyopathy [95,96]. These studies, and numerous others described in several outstanding review articles [97,98], present compelling evidence of defects in peripheral tolerance, suggesting a regulatory role for the PD pathway under homeostatic conditions. Enthusiasm for investigating these molecules has increased steadily with the revelation that not only can this immune checkpoint be inhibited with neutralizing monoclonal antibodies, but that inhibition of PD-PD-L signaling can improve survival in human cancer patients as well as in numerous murine cancer models. Murine models have shown that blocking any of PD-1 [99,100], PD-L1 [100,101], or PD-L2 [101] has beneficial effects in inhibiting the growth and spread of tumors; conversely, constitutive expression of PD-L1 by tumor cells leads to enhanced resistance to CD8^+^ T cell-mediated cytolysis [102]. In a series of exciting developments, inhibitors of the PD-1 signaling pathway have shown considerable efficacy in numerous clinical trials involving patients with malignant disease who have failed conventional therapies (reviewed in [103,104]). Thus, in addition to understanding the effects of the PD-1 signaling pathway on fungal pathogenesis (reviewed below), it will be essential to ascertain whether the expanding clinical use of checkpoint inhibitors will alter the susceptibility or severity of fungal infections in patients treated with these immunotherapy agents.

The immunomodulatory properties of the PD-1 signaling pathway in response to infectious pathogens were initially characterized in the context of chronic viral infections (refer to several appropriate reviews [105,106]). Additional studies have defined a role for this pathway in response to numerous non-fungal pathogens [107,108,109]. Despite a relative paucity of studies investigating the PD signaling pathway in the context of fungal disease, the pathway has been studied in select fungal infections with results suggesting a major role for the pathway in perpetuating persistent fungal disease. Expression of both PD-L1 and PD-L2 increases alveolar macrophages (AMs) during the course of pulmonary infection with *C. neoformans* [110] and *H. capsulatum* [111]. T cells obtained from patients with paracoccidiomycosis over-express PD-1 as compared to T cells from healthy volunteers, whereas other similar molecules (e.g., CD28) had unchanged expression [112]. However, in contrast to other studies, the authors of this study found that neither anti-PD-1 nor anti-PD-L1 antibody treatment restored proliferative capacity of T cells from infected donors in vitro, which suggested the possibility of other, possibly redundant, pathways affecting the proliferative capacity of T cells.

In addition to its effects on T cells, it is important to recognize that the PD-1/PD-L signaling pathway is bidirectional with additional effects on the cognate PD-L expressing APCs. DCs acquire a suppressive phenotype when exposed to soluble PD-1 (sPD-1) as evidenced by their reduced expression of costimulatory molecules CD40, CD80, and CD86, and increased production of IL-10 relative to isotype antibody-exposed DCs [113]. Although studies directly examining this ‘reverse signaling’ phenomenon in fungal infections are lacking, this is an area in need of further research due to its implications for utilizing antibodies as treatment. Also of interest is the potential for the PD-1 signaling pathway to mediate immunoregulatory interactions between APCs. This was demonstrated in a murine model of *Pneumocystis* pneumonia (PcP) in which myeloid-derived suppressor cells (MDSCs) were found to accumulate in the lungs of infected mice and cause subsequent lung damage. These MDSCs expressed high levels of PD-L1, whereas resident alveolar macrophages expressed increased amounts of PD-1 in response to infection. The authors showed that in vitro co-culture of alveolar macrophages obtained from uninfected mice with MDSCs from PcP mice resulted in an 18-fold increase in PD-1 expression associated with a significant impairment in the phagocytic capacity of these macrophages. Critically, addition of an anti-PD-L1 antibody reduced these effects [93], demonstrating that the PD signaling axis was specifically modulating AM phagocytic function, emphasizing that interactions between myeloid cells can modulate their respective phenotypes via PD-1 signaling.

Fungal sepsis represents an acute form of disseminated candidiasis that may require more rapid immunoregulation than many chronic infections such as pulmonary cryptococcosis and paracoccidiomycosis. Patients suffering from *C. albicans* fungal sepsis display elevated levels of PD-1 on CD8^+^ T cells, as well as increased expression of PD-L1 on both CD4^+^ and CD8^+^ T cells [114]. In this study, the control group was critically ill non-septic patients with no evidence of fungal infection, suggesting that septic infection with the fungal pathogen *C. albicans* was an important and specific determinant in activation of the PD signaling pathway. Using both one- and two-hit fungal sepsis models, Chang et al [115] demonstrated that blockade of PD signaling utilizing either anti-PD-1 or anti-PD-L1 antibodies significantly improved mouse survival in both models. PD-1 expression was found to be upregulated on both CD4^+^ and CD8^+^ T cells as early as 3 days post-infection, demonstrating that PD-1 upregulation is an important early physiological response to an acute fungal infection. Addition of anti-PD-1 antibody also led to significant increases in splenocyte secretion of IFNγ, IL-10, and IL-6, and increased expression of MHC II on both macrophages and dendritic cells, further supporting a role for PD-1 in inhibiting a broad array of immune activity. Thus, these studies identify an important and potentially unique role for the PD-1 signaling pathway in response to an acute and systemic fungal infection resulting in sepsis.

A lethal murine model of *H. capsulatum* infection provides additional exciting evidence for an important role of PD signaling in regulating immunity toward an acute fungal pathogen [111]. Whereas 100% of WT mice succumbed to disease within 4 weeks of initial infection, 100% of PD-1^−/−^ mice survived disease free for at least 90 days. Of note, although both WT and PD-1^−/−^ mice developed disseminated disease, this trend was rapidly reversed in PD-1^−/−^ mice as *H. capsulatum* was completely eradicated from all organs by 13 days post infection in these animals. The authors demonstrated that PD-L1 and PD-L2 were both up-regulated on lung macrophages and DCs, and these cells significantly impaired both CD4^+^ and CD8^+^ T cell proliferation in vitro. To more accurately reflect therapeutic potential, the authors also blocked the PD-1 signaling pathway (using an anti-PD-1 neutralizing antibody) during infection of WT mice, and found a 70% survival rate that persisted for at least 6 months, as compared to 0% survival seen in the untreated group. Our preliminary studies using a murine model of persistent cryptococcal lung infection support and extend these findings as we have shown that treatment of mice with an anti-PD-1 blocking antibody to mice with established infection enhances T cell activation and reduces fungal burden (unpublished data). The results of these studies raise the exciting possibility that immune checkpoint inhibitors may represent novel immunotherapeutics for the treatment of chronic lung infections.

In summary, an emerging body of literature identifies the PD-1 signaling pathway as a unique immunoregulatory pathway capable of mediating bidirectional effects amongst myeloid cells and between myeloid cells and T cells in response to fungal infections. As an immune checkpoint, this pathway may represent a novel target for applied therapeutics designed to enhance immunity against persistent fungal diseases that can be difficult to treat with antibiotics alone. Thus far, there is no evidence that the use of checkpoint inhibitors in the treatment of patients with cancer alters their susceptibility to fungal infections or triggers IRIS (reviewed below) yet these possibilities warrant ongoing monitoring.

### 3.3. Cytotoxic T Lymphocyte-Associated Protein 4 Signaling

Similar to PD-1, CTLA-4 has been shown to regulate T cell activation in response to tissue grafts, demonstrating a valuable effect on peripheral tolerance, as CTLA-4 blockade accelerates tissue graft rejection [116]. Unlike PD-1, which has its own unique ligands, CTLA-4 binds to the more well-studied costimulatory molecules CD80 and CD86 [117,118,119], subsequently acting to inhibit T cell activation and effector function [120,121,122]. In this way, CTLA-4 competes with CD28 and not only reduces costimulatory signaling via CD80 and CD86 binding to CD28, but actively inhibits T cell activity upon binding these ligands [123,124]. Under some circumstances, CTLA-4 ligation can override CD28-dependent T cell activation, although IL-2 can in turn override the effect of CTLA-4 and restore activation [125]. Thus, CTLA-4 can be viewed as having an effect opposite that of CD28 [126], as compared to the relatively unique signaling seen within the PD-1 axis. This distinction is supported by data suggesting a synergistic, rather than redundant, role for the two pathways. Loss of PD-1 results in priming of autoreactive CD8^+^ T cells in a mouse model of peripheral CD8^+^ T cell tolerance; blocking CTLA-4 signaling has a similar effect. When both pathways are blocked, however, the effect is enhanced, suggesting functional non-redundancy [127]. Although both pathways were not blocked simultaneously, a study utilizing a murine model of *C. albicans*-induced fungal sepsis showed that blocking signaling through PD-1, PD-L1, or CTLA-4 resulted in similar improvements in survival, demonstrating a comparable magnitude of effect between the two pathways during candidiasis [115].

As with PD-1, research into the impact of CTLA-4 signaling on regulation of immune responses to fungal diseases is limited but promising. Studies performed on patients with PCM have demonstrated increased CTLA-4 activity relative to healthy patients or patients receiving treatment [67], leading the authors to speculate that increased CTLA-4 activity contributes to the relative immunosuppression known to occur in this disease, perhaps by mechanisms related to CTLA-4 induced apoptosis of T cells. Alternatively (or in addition), this study also showed that simultaneous blockade of CTLA-4 and Fas ligand on T cells from PCM patients resulted in enhanced T cell proliferation in vitro; thus, inhibition of T cell proliferation might be an additional mechanism of CTLA-4 mediated immune regulation in patients with PCM [128].

Investigations of *C. neoformans* infection have provided additional insights into the inter-relationship of this signaling pathway with cryptococcal virulence factors and the ability to establish effective immune responses following vaccination. Specifically, murine CD4^+^ T cells stimulated with *C. neoformans* up-regulate CTLA-4 expression rapidly after exposure. Further, *C. neoformans*-stimulated CD4^+^ T cells proliferate and produce cytokines including IL-2 and IFNγ in response to CTLA-4 blockade, as compared to stimulated CD4^+^ T cells in the absence of blocking anti-CTLA-4 antibodies [129]. The authors further noted differences in CTLA-4 up-regulation when cells were stimulated with virulent, capsular *C. neoformans* as compared to the relatively benign acapsular form. Thus, induction of CTLA-4 may be one means of immune evasion used by some fungal pathogens. Efforts to counteract CTLA-4-mediated immune evasion may represent an effective therapeutic strategy as supported by one promising study [130], which demonstrated that CTLA-4 blockade enhanced survival of mice infected with a highly virulent strain of *C. neoformans*. Enhanced survival was mirrored by reduced fungal burden in the lung, spleen, and brain, demonstrating the potential of CTLA-4 blockade to both inhibit fungal growth at the initial site of infection and to limit potentially lethal dissemination. Perhaps most intriguing, however, was the additional observation that administration of anti-CTLA-4 antibodies during induction of a cell-mediated immune response to *C. neoformans* by vaccination improved the efficacy of vaccination and increased protection against subsequent infection with the organism. These data support and extend findings from a study demonstrating increased CTLA-4 expression on Th17 cells relative to Th1 cells in response to *C. albicans* infection [131]. Together, these studies may guide future vaccination strategies designed to elicit more potent recall responses from specific T cell subsets when traditional vaccination is combined with anti-CTLA-4 antibody treatment.

## 4. Unique Immunoregulatory Circumstances

### 4.1. Protective Tolerance

Protective tolerance represents a host preservation immune strategy that functions to limit immunopathology while controlling an infection. This strategy appears especially pertinent to fungal infections given their propensity to evade initial host defenses, which often leads to lengthy and persistent attempts by the host to achieve sterilizing immunity at the risk of incurring host tissue damage. As such, in some instances it is advantageous for the host to contain and coexist with a (fungal) pathogen rather than continuously striving to attain absolute fungal clearance. Protective tolerance may also be viewed as the development of commensalism between host and fungus [132], born out of necessity due to the ubiquitous nature of many fungi in the environment.

Both regulatory T cells and IL-10 are prominent contributors to protective tolerance. Studies investigating oral tolerance to *C. albicans* have shown that the disruption of regulatory pathways involving CD28, CD86, and IL-10 leads to an enhanced ability to restrict fungal growth but at the cost of inflammatory immunopathology [133]. As *C. albicans* is a well-established commensal within the oral microbiome, protective tolerance would seem evolutionarily favorable rather than unleashing the full capabilities of host defenses to eradicate the organism given the “collateral damage” caused by such efforts. Similarly, although not typically viewed as a commensal microorganism, *A. fumigatus* can establish persistent infection and ultimately necessitate the development of protective tolerance for the well-being of the host. Data demonstrating that DCs activate Treg cells [134,135] and dampen inflammatory Th1 and Th17 responses to the fungus [134,136] provide evidence that the host may avail itself to this strategy when encountering this organism. Although not directly implicated in protective tolerance against *C. albicans* or *A. fumigatus*, the involvement of the CD28-CD80/86 signaling axis suggests that the CTLA-4 and perhaps the PD-1 signaling pathways might also be involved; further investigation into the role of these pathways in protective tolerance is warranted.

Collectively, the concept of protective tolerance can be viewed as a unifying theme with regard to the immunoregulatory mechanisms discussed within this review. Protective tolerance, while a relatively novel concept in need of further investigation, likely relies on several or all of the immunoregulatory mechanisms discussed above to function properly. Evidence suggests that Treg cells are a critical mediator of protective tolerance [71,137]. Dendritic cells promote the development and activity of Treg cells, in part through production of IL-10, and Treg cells in turn utilize IL-10 to influence both innate and adaptive immunity. Treg cells also utilize PD-1 and CTLA-4 signaling to exert their immunoregulatory effects and are thus identified as a central regulatory cell through which several other immunoregulatory mechanisms mediate their immunomodulatory effects. Thus, protective tolerance results from the cumulative effects of several immunoregulatory mechanisms working in concert to effectively control fungal infections while limiting host damage, thereby resulting in commensalism.

### 4.2. Immune Restoration Inflammatory Syndrome

As the focus of this review is mechanisms of immunoregulation in fungal disease, much of the discussion has centered on studies in which these regulatory processes dampen inflammation. Emerging evidence of a unique phenomenon termed Immune Restoration Inflammatory Syndrome (IRIS), a syndrome of excessive inflammation in response to rapid immune reconstitution, raises a red flag to researchers regarding the potential drawbacks of interfering with these tightly-controlled pathways. Shelburne and colleagues proposed the following clinical definition of IRIS: “a paradoxical deterioration in clinical status attributable to the recovery of the immune system during highly active antiretroviral therapy (HAART) of HIV infection” [138]. As will be discussed, IRIS is not limited to individuals undergoing HAART, but most instances of IRIS are seen in this context. A rapid increase in the number of CD4^+^ T cells present likely plays a critical role in the development of IRIS, as evidence suggests that a high baseline CD4^+^ T cell count is protective against developing IRIS, and conversely lower CD4^+^ T cell counts are predictive of IRIS development [139,140]. Thus, IRIS occurs while adaptive immunity is being restored following immunosuppression [141,142].

Although some studies show that HAART patients experiencing IRIS tend to have a slightly better long-term prognosis than those that do not (presumably due to the host’s robust immune system providing a greater long-term benefit), one cannot discount the significant short- and medium-term morbidity associated with the disease, as IRIS symptoms can persist for two years following immune restoration [140]. IRIS is a significant concern for HIV^+^ individuals initiating HAART, as reinvigorated immune responses to latent or previously controlled infections may lead to widespread immunopathology in the host. There are two broad categories of IRIS, unmasking and paradoxical. Unmasking IRIS occurs when a previously unknown opportunistic pathogen is present for which a patient had previously tested negative but tests positive upon initiation of HAART, with concomitant development of symptoms. Paradoxical IRIS occurs when a disease has been previously diagnosed and the patient received treatment prior to the initiation of HAART, with the patient experiencing symptoms of IRIS associated with inflammatory responses to the infection [139].

Indeed, whereas *M. tuberculosis* represents the most common pathogen causing IRIS, primary or coinfection with *C. neoformans* is also exceedingly common in IRIS patients [143,144]. Current estimates vary, but anywhere from 8%–43% of HIV^+^ patients previously treated for tuberculosis, and 4%–66% of those previously treated for cryptococcosis, undergoing HAART, develop symptoms of IRIS following initiation of treatment [145]. IRIS is particularly relevant to immune dysregulation in the context of fungal infections, as much research into this syndrome focuses on individuals with active cryptococcosis or latent *C. neoformans* infection. Cryptococcal IRIS is potentially life-threatening [146], as fungal antigens residing within the central nervous system (CNS) can trigger lethal IRIS-associated excessive inflammation in this location. Risk factors for cryptococcal IRIS include high baseline fungal burden, an ineffective host response to the initial infection, and a subsequent rapid restoration of immunity (e.g., due to HAART) [147].

Despite the elegant immunomodulatory networks described in our discussion of protective tolerance, there is evidence that many of these regulatory mechanisms also contribute to IRIS [148]; in addition, there is evidence suggesting that many of the pathogens promoting IRIS are of fungal origin. Cryptococcal IRIS is associated with a skewing of the immune system from a Th2 response to a Th1 response [147] associated with additional increases in Th17 and natural killer (NK) cell responses and elevated production of inflammatory cytokines including IL-6, IL-7, and IFNγ [148,149]. One study identified a 10-fold increase in serum IFNγ and IgG in cryptococcal meningitis (CM) IRIS patients on HAART as compared to HAART patients without CM-IRIS or healthy controls. Interestingly, the authors also noted an increase in the abundance of Treg cells within these individuals, suggesting a role for these regulatory cells in the development or suppression of IRIS [150]. The most precise estimates of IRIS occurrence range from 25%–32.5%, developing on average 8 weeks after the initiation of HAART, with increased expression of pro-inflammatory markers including IFNγ, TNFα, and eotaxin in CM-IRIS patients as compared to individuals simply experiencing a relapse of CM [151,152]. This distinction is critical, as it clearly shows that IRIS is an immunological phenomenon distinct from basic host immune responses to *C. neoformans* in the CNS.

Although IRIS is typically viewed from the perspective of HIV patients undergoing HAART, other circumstances associated with restoration of host defenses are capable of triggering IRIS. IRIS has been shown to occur in roughly 5% of *C. neoformans*-infected transplant patients and 14% of *M. tuberculosis*-infected transplant patients upon cessation of immunosuppressive therapy [153,154]. One critical distinction, however, is that whereas HAART-IRIS patients typically experience a clinical deterioration followed by recovery, transplantation IRIS greatly increases the risk of allograft rejection, thus severely reducing the chances of recovery. *C. neoformans*-induced transplantation IRIS in particular has been shown to cause a significantly higher incidence of graft rejection, with studies demonstrating 2–11 times greater rejection frequency [153,155]. Critically, approximately 54%–72% of these cases involve dissemination to the CNS, thus further increasing the likelihood of morbidity and mortality [155]. Although *C. neoformans* is the most commonly observed fungal infection during transplantation IRIS, invasive aspergillosis has also been demonstrated to trigger IRIS in transplant recipients [156]. Interestingly, McLin and colleagues have recently proposed that pediatric transplantation patients may be protected from IRIS. Specifically, the authors suggest that the relative absence of IRIS in pediatric patients may be attributable to thymus-dependent immune reconstitution, which may promote the generation of more Treg cells, thereby creating a more balanced restoration of immunity relative to the less diverse and more highly polarized lymphocyte reconstitution that occurs in adults. Although intriguing, the suggestion is speculative, underscoring the need for additional research in this field [157].

In addition to cases in HAART and transplantation patients, IRIS has been observed in women post-parturition; this has been shown due to infection with both *C. neoformans* [158,159,160] and *Coccidioides immitis* [161,162]. Although pregnancy is generally only mildly immunosuppressive, the dysregulated reconstitution of immunity following childbirth is sufficient to induce IRIS. During pregnancy, in addition to general immunosuppression, the immunological environment shifts in favor of Th2 immunity, which in turn provides a more hospitable environment for the establishment of fungal infections or reactivation of latent infections [163,164,165]. A reversal in this balance following childbirth, when Th1 immunity becomes more pronounced, has been documented [166], thus providing one means by which IRIS may be triggered in the post-partum period.

In summary, protective tolerance and IRIS can best be viewed as opposite outcomes along the spectrum of immunoregulatory mechanisms active in response to fungal infections. Both scenarios identify the complex and often complementary mechanisms utilized by the immune system in its efforts to achieve healthy immune homeostasis. Our increased understanding of these mechanisms may allow us to prevent or better treat the morbidity and mortality that may result when these mechanisms become dysregulated.

## 5. Conclusions

Fungal infections present unique challenges to the host immune system. As fungi are more similar to mammals than are other pathogens such as bacteria or viruses, sterilizing immunity is often difficult to achieve, and conventional treatments are far more limited due to the greater potential for damage to the host. This in turn can lead to prolonged infections leaving the host at increased risk for immunopathology due to ongoing inflammatory processes in response to the pathogen. Ultimately, immunoregulatory mechanisms are required to minimize host damage while simultaneously allowing effective immune responses to continue. Immunoregulation is a double-edged sword, however, as a reduction in Th1 and pro-inflammatory processes can allow pathogens to multiply and potentially spread to secondary locations such as the CNS.

In this review, we have highlighted several cell types and pathways activated in response to fungal infections (summarized in Table 1). We have identified dendritic cells and regulatory T cells as crucial regulatory immune cells that orchestrate both innate and adaptive immunity through direct and indirect mechanisms. Although multiple immune processes contribute to immune regulation in response to fungal infections, we focused our attention on the IL-10, PD-1, and CTLA-4 signaling pathways as individually and collectively they have proven to be of central importance to numerous immunoregulatory networks. Working in concert, these cells and pathways may comprise an effective strategy to establish commensal relationships with fungi through protective tolerance. In contrast, dysregulation amongst these cells and pathways may result in over-exuberant and deleterious inflammation as observed in IRIS. Perhaps most exciting are the opportunities that we have highlighted in which these cells and pathways might be intentionally manipulated to enhance our ability to prevent or treat fungal disease. Such advances will require the continued investment of our scientific community including ongoing partnerships with basic scientists, clinicians, and pharmaceutical companies.

## Figures and Tables

**Table 1:** Concepts Relevant to Immunomodulation in Fungal Disease.

Concept	Specific Topic	Fungi of Relevance	References
Cells	Dendritic Cells	*A. fumigatus*	[20,21,35]
		*C. albicans*	[28,29,68]
		*C. neoformans*	[9,30,31,32,33,36,37,38]
		*H. capsulatum*	[34]
	Regulatory T Cells	*A. fumigatus*	[64]
		*C. albicans*	[58,59,65,67,68,69,71]
		*C. neoformans*	[61,62]
		*H. capsulatum*	[63]
		*P. brasiliensis*	[60,66,67]
Signaling Pathways	IL-10	*A. fumigatus*	[84,87]
		*C. albicans*	[83,85]
		*C. neoformans*	[9,36,37,38,39,81]
		*H. capsulatum*	[86]
	PD-1	*C. albicans*	[114,115]
		*C. neoformans*	[110]
		*H. capsulatum*	[111]
		*P. brasiliensis*	[112]
		*P. jirovecii*	[93]
	CTLA-4	*C. albicans*	[115,131]
		*C. neoformans*	[129,130]
		*P. brasiliensis*	[67,128]
Unique Circumstances	Protective Tolerance	*A. fumigatus*	[134,135,136]
		*C. albicans*	[71,133]
	Immune Restoration Inflammatory Syndrome	*A. fumigatus*	[156,157]
		*C. immitis*	[162,163]
		*C. neoformans*	[143,144,145,146,147,148,149,150,151,152,153,155,159,160,161]
